# Genome-Wide Association Study of Genetic Control of Seed Fatty Acid Biosynthesis in *Brassica napus*

**DOI:** 10.3389/fpls.2016.02062

**Published:** 2017-01-20

**Authors:** Katarzyna Gacek, Philipp E. Bayer, Iwona Bartkowiak-Broda, Laurencja Szala, Jan Bocianowski, David Edwards, Jacqueline Batley

**Affiliations:** ^1^Plant Breeding and Acclimatization Institute—National Research Institute, Oilseed Crops Research CentrePoznan, Poland; ^2^School of Plant Biology, University of Western AustraliaPerth, WA, Australia; ^3^Department of Mathematical and Statistical Methods, Poznan University of Life SciencesPoznan, Poland

**Keywords:** *Brassica napus*, skim genotyping by sequencing (Skim GBS), GWAS, fatty acid biosynthesis, seed quality

## Abstract

Fatty acids and their composition in seeds determine oil value for nutritional or industrial purposes and also affect seed germination as well as seedling establishment. To better understand the genetic basis of seed fatty acid biosynthesis in oilseed rape (*Brassica napus* L.) we applied a genome-wide association study, using 91,205 single nucleotide polymorphisms (SNPs) characterized across a mapping population with high-resolution skim genotyping by sequencing (SkimGBS). We identified a cluster of loci on chromosome A05 associated with oleic and linoleic seed fatty acids. The delineated genomic region contained orthologs of the *Arabidopsis thaliana* genes known to play a role in regulation of seed fatty acid biosynthesis such as Fatty acyl-ACP thioesterase B (*FATB*) and Fatty Acid Desaturase (*FAD5*). This approach allowed us to identify potential functional genes regulating fatty acid composition in this important oil producing crop and demonstrates that this approach can be used as a powerful tool for dissecting complex traits for *B. napus* improvement programs.

## Introduction

Oilseed rape (*Brassica napus* L.) is now the second largest oil-producing crop in the world after soybean (70.9 and 314.5 million tons respectively; FAO^1^). Three of the major fatty acids (FAs) in oilseed rape oil are the monounsaturated FA oleic acid and the two polyunsaturated FAs linoleic acid and linolenic acid (Smooker et al., 2011). The high nutritional value of *B. napus* oil compared to other vegetable oils is attributed to a high level of oleic acid, as well as an optimal ratio (2:1) of the essential linoleic (omega 6) and linolenic (omega 3) FAs (Hu et al., 2006). For industrial purposes, thermal stability of *B. napus* oil is assured with a low level of linolenic acid. Understanding the genetic basis of fatty acid biosynthesis in oilseed rape is of great importance in order to manipulate its content.

The process of oil biosynthesis has been well-characterized in the model species and closely related member of Brasicaceae; *Arabidopsis thaliana*. This complex process employs a coordinated action of genes involved in seed maturation, energy metabolism, fatty acid, and triacylglycerol (TAG) biosynthesis pathways (Baud and Lepiniec, 2010; Bates et al., 2013). *De novo* synthesis of FAs occurs within plastids of the seed and enables formation of palmitic acid (16:0), stearic acid (18:0), and oleic acid (18:1). Once produced, they are released by two classes of acyl-ACP thioesterase (FAT) enzymes: FATA with higher affinity to 18:1-ACP and FATB with higher affinity to 16:0-ACP (Bonaventure et al., 2003). Plastidial FAs are transported to the endoplasmic reticulum (ER) where they can undergo desaturation modification via FAD2 and FAD3, key enzymes known to generate polyunsaturated linoleic (18:2) and linolenic (18:3) FAs respectively (Okuley et al., 1994; Yang et al., 2012). In parallel to a desaturation pathway, FAs can be elongated by *FAE1* to erucic acid or esterified to glycerol to generate TAG, a major form of seed oil in plants (James et al., 1995). Although the metabolic pathways for fatty acid biosynthesis synthesis are well known, genetic regulation of these pathways, and thus variable fatty acid composition in seed, is still poorly understood.

*B. napus* (AACC, 2*n* = 38) originated from natural hybridization between *B. rapa* (AA, 2*n* = 20) and *B. oleracea* (CC, 2*n* = 18) around 7500 years ago. The diploid progenitors of *B. napus* underwent genetic triplication which led to formation of large gene families and abundant repetitive sequences. Oil biosynthesis genes have undergone expansion in *B. napus* which exceeds that known in other oilseed plants (Chalhoub et al., 2014). Polyploidy and the genome complexity of *B. napus* limit translation of fundamental knowledge from *A. thaliana* into oilseed rape crop improvement as identification of individual genes controlling natural variation in this crop is challenging (Wells et al., 2014).

To date, several studies have been undertaken to dissect the genetic architecture of fatty acid biosynthesis in oil crops. Quantitative trait loci (QTL) mapping studies in *B. rapa* (Basnet et al., 2016), *Glycine max* (Wang et al., 2014), *Jatropha curcas*, and *B. napus* allowed identification of loci with small to large allelic effect involved in fatty acid biosynthesis in seeds (Burns et al., 2003; Hu et al., 2006; Zhao et al., 2008; Yan et al., 2011; Wang et al., 2015). Orthologs encoding major enzymes involved in FA biosynthesis, such as *FAD2* and *FAD3*, have been mapped in *B. napus* on chromosomes A1, A5, C1, and C5 (Scheffler et al., 1997; Schierholt et al., 2000; Yang et al., 2012) and A3, A4, A5, C3, and C4 (Hu et al., 2006; Smooker et al., 2011) respectively. Recently, a systems genetic approach that combined gene expression studies with QTL genetic mapping (eQTL) led to identification of other *FAD* genes (*BrFAD5* and *BrFAD7*) playing an interactive role with *BrFAD2* in regulation of oleic and linoleic FAs in *B. rapa* (Basnet et al., 2016). Transcriptional analysis studies in developing seeds of Arabidopsis and *B. napus* showed that regulation of FA biosynthesis is complex and involves genes responsible for transcriptional regulation, starch metabolism, as well as auxin and jasmonate hormone signaling (Niu et al., 2009; Mendes et al., 2013; Chen et al., 2015).

Recently, genome wide association studies (GWAS) have evolved as a powerful tool to dissect the genetic architecture of complex traits in crop species (Edwards et al., 2013). Advances in next generation sequencing (NGS) allow identification of thousands of genetic marker loci which enables their statistical association with traits of interest based on linkage disequilibrium (Davey et al., 2011). Skim-based genotyping by sequencing (skimGBS) uses low-coverage (1–10x) whole genome sequencing for high resolution genotyping. Genomic reads from parental individuals are mapped to the reference genome and SNPs are predicted. Reads from the progeny are then mapped to the same reference and comparison with the parental SNP file enables the calling of SNPs in the progeny of one or other of the parental genotypes (Bayer et al., 2015). Associated genetic markers can be causal for the trait of interest or in linkage disequilibrium with a causal locus (Rafalski, 2010). To date, GWAS approaches using whole genome sequencing have allowed researchers to dissect genetic regulation of complex traits such as oil biosynthesis, carotenoid concentration and yield in well studied crops including maize and rice (Gao et al., 2013; Li H. et al., 2013; Suwarno et al., 2015). In oilseed rape, GWAS using DartSeq and Brassica 60K SNP array genotyping approaches allowed identification of alleles involved in regulation of flowering time, as well as seed quality traits including germination, vigor and seed weight (Li et al., 2014; Hatzig et al., 2015; Raman et al., 2016).

The aim of the present study was to perform GWAS association mapping using whole genome SkimGBS (Bayer et al., 2015) to identify allelic variation that affects fatty acid composition in progeny seeds from 60 *B. napus* doubled haploid (DH) lines. This novel approach led to identification of a genomic hotspot of candidate regulatory genes on chromosome A05 of winter type oilseed rape.

## Materials and methods

### Plant material and growth conditions

A *B. napus* DH mapping population of 60 lines, developed from a cross between recombinant inbred lines RIL324, as a female parent, and RIL622, as a male parent, was used in this study. The two parents represent winter oil seed rape canola type and differ in fatty acid content. The phenotypic experiments were carried out in 2013/2014 using randomized block experimental design. Seeds of the DH lines and parents were germinated on standard soil mixture and grown in a controlled environment room until a 3–4 leaf stage. To allow vernalization, the plants were placed in a cold room (4°C) for 7 weeks. Two plants per DH line were transplanted to plastic pots filled with standard soil mixture and grown in the glasshouse until maturity. Individual DH lines were grown in four replicates (four plants) whereas the parental lines were grown in ten replicates. Dry mature seeds were harvested from each from each of the replicated plant and used for fatty acid measurements.

### Fatty acid measurement

Seed oil was analyzed for fatty acid composition using a gas chromatograph fatty acid methyl esters (GC FAME) method. Transmethylation of the extracted lipids was performed with 0.5 M KOH in methanol for 15 min at 70 °C, followed by the extraction of fatty acids as methyl esters in hexane. Fatty acid composition was determined with an Agilent 6890 gas chromatograph, column 30 m DB25, 200 °C and flame ionization detector (FID) (Michalski, 2006). The statistical analysis of traits and regression analysis of candidate SNPs was performed using Genstat.

### Genotyping by sequencing (GBS): skim based genotyping

Genomic DNA (gDNA) from the individual plants within the lines was extracted using a standard Doyle/CTAB method (Doyle and Doyle, 1987). To check the DNA quality samples were visualized using agarose gel electrophoresis followed by quantification of the samples using the Qubit fluorometric method. TruSeq Nano DNA Libraries were prepared for all samples according the manufacturer's instructions (Illumina®). The library insert size was 500 bp. Paired end skim GBS data (150 bp) was produced using the Hiseq 2500 at the Australian Genome Research Facility (AGRF).

SNPs and genotypes were called using SGSautoSNP and the SkimGBS pipeline (Lorenc et al., 2012; Bayer et al., 2015). Skim-based genotyping by sequencing (skimGBS) uses low-coverage (1–10x) whole genome sequencing and is a two-stage method that requires a reference genome sequence, genomic reads from parental individuals and individuals of the population. In this study reads were mapped to the *B. napus* Darmor reference (Chalhoub et al., 2014) using SOAPaligner/soap2 v2.21 (Li et al., 2009) (options: Insert size 0–1000, report reads aligning non-repetitively). Subsequent mapping of the progeny reads to the same reference and comparison with the parental SNP file enables the calling of the parental genotype. According to the SGSautoSNP protocol, read data were not trimmed or filtered (Bayer et al., 2015).

### Genome-wide association study

Genome-wide association was performed using GAPIT with standard settings and 2 PCAs (Lipka et al., 2012). The selection of candidate genes was based on the subset of SNPs with the lowest *p*-values after FDR-correction (standard GAPIT FDR adjustment). Genes within ~50 kb upstream and downstream to associated SNPs were analyzed for annotation, and those with allelic variation between the parents were selected as plausible candidate genes.

### *In silico* mapping of SNPs

Physical mapping of significantly associated SNPs and functional annotation of the predicted genes harboring these SNPs was performed using the *B. napus* genome browser (http://www.genoscope.cns.fr/brassicanapus/) (Chalhoub et al., 2014). Kyoto Encyclopedia of Genes and Genome (KEGG) (http://www.genome.jp/kegg/genes.html) and Acyl-Lipid Metabolism (http://aralip.plantbiology.msu.edu/pathways/pathways) were used in this study to identify genes that play a role in lipid metabolism, Prediction of SNP variation and effect was performed with SnpEff using the public Darmor annotation v5 (Cingolani et al., 2012).

### SNP validation

In order to validate the accuracy of SNP prediction and phenotypic association mis-sense SNPs in three of the candidate genes identified through GWAS or close to linked SNPs were PCR amplified and sequenced. PCR primers flanking the SNP were designed (**Table 4**) using PRIMER3 software (Rozen and Skaletsky, 2000). All PCR reactions were carried out in 50 μl reaction volumes containing 40 ng of template DNA, 25 μl Phusion Hot Start II High Fidelity (Thermo Scientific, Australia) master mix and 0.5 μM of forward and reverse primer. Cycling conditions consisted of an initial hot start at 98°C for 30 s; 33 cycles of denaturation at 98°C for 10 s, annealing at 60–64°C for 20 s and extension at 72°C for 30 s. The reaction was completed with a final extension cycle at 72°C for 5 min. Post-PCR purification of the PCR fragments was carried out using Ampure XP beads (Beckman Coulter,) following the manufacturer's recommendations. Purified PCR fragments were submitted to the Australian Genome Researh Facility for Sanger sequencing. Sequencing data was trimmed and subsequently aligned using Geneious R10 using Global Alignment with free end gaps (Cost matrix: 93% similarity (5.0/-9026168). Aligned sequences were compared with the reference and alternative sequence to verify the SNPs.

### GWAS validation

In order to validate the associated regions identified through GWAS analysis, QTL mapping was performed. SNPs of the population were imputed using LD-kNNi (Money et al., 2015) as implemented in TASSEL v5.2.30 (Glaubitz et al., 2014). MSTMap (Wu et al., 2008) was used to create a genetic map with the options: Cut_off_*p*_value 2, no_map_size 2, no_map_dist 15, missing_threshold 0.25). R/qtl v 1.40-8 (Broman et al., 2003) and R v3.3.1 (R Core Team, 2016) were used to calculate LOD thresholds and LOD scores for all phenotypes using the MSTMap results as positions (calc.genoprob with options step = 1 and error.prob = 0.001, scanone with n.perm = 1000 to calculate LOD thresholds and without n.perm to calculate LOD scores).

## Results

### Phenotypic variation correlations of fatty acids in seeds/traits

**Figure 1:**
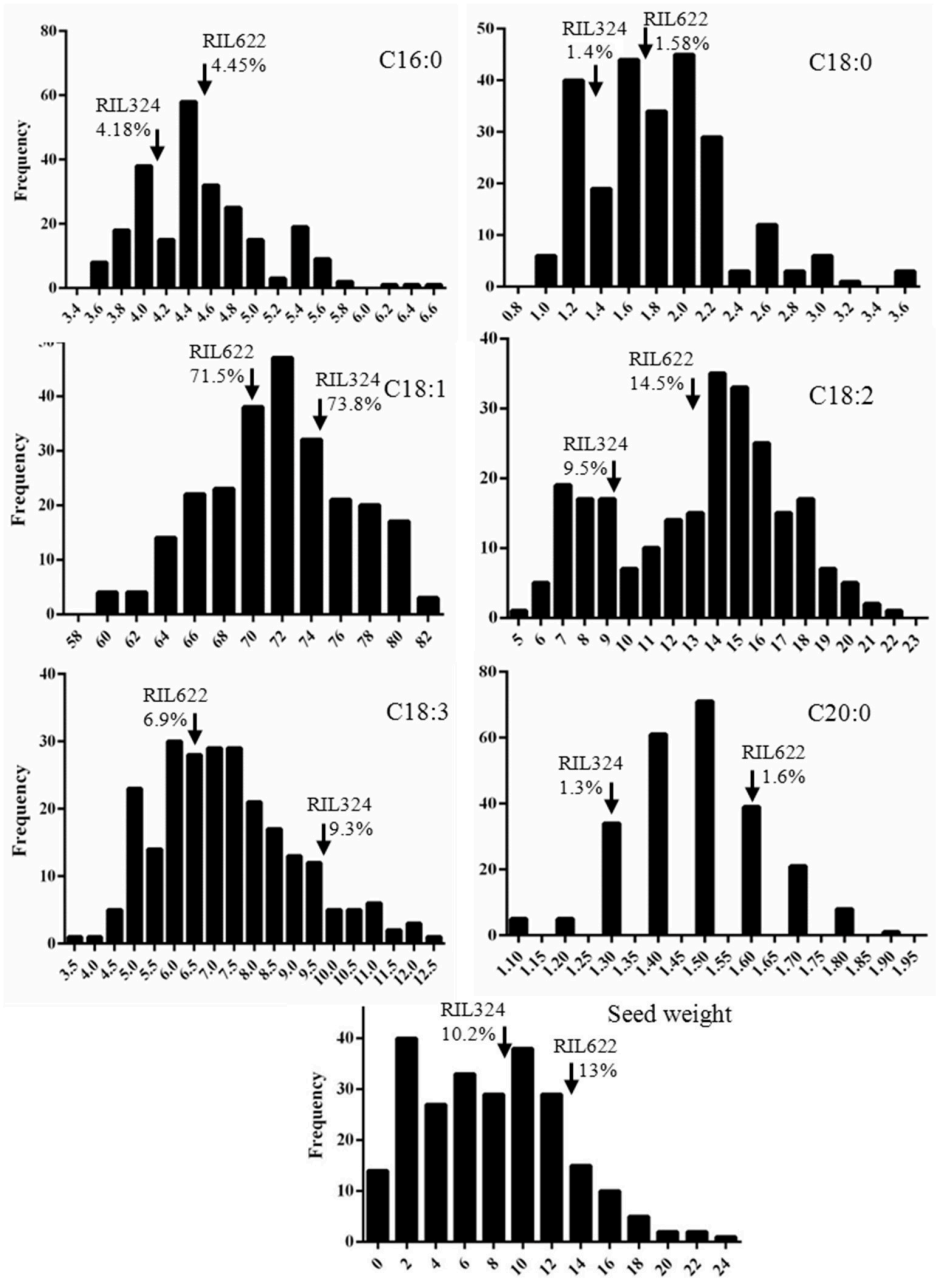
**Distribution of fatty acid concentrations in RIL324xRIL622 DH mapping population**. Distribution of palmitic (C16:0), stearic (C18:0), oleic (C18:1), linoleic (C18:2), and linolenic (C18:3) fatty acid and seed weight in the RIL324xRIL622 *B. napus* mapping population lines. Fatty acids were measured as a percentage of the total oil content, mean values are represented as a frequency within the mapping population. Arrows represent fatty acid level in parental lines RIL324 and RIL622.

Palmitic C16:0, stearic (C18:0), oleic (C18:1), linoleic (C18:2), linolenic (C18:3) fatty acids in the total oil content and seed weight were measured for four replicates in each of the 60 DH lines of the *B. napus* panel (Tables 1, 2). The population displayed normal, or near-normal, distribution for C16:0, C18:0, C18:1, C18:3, and C20:0 acids, that suggests complexity of their genetic networks (Wang and Ruan, 2012). C18:2 showed a bi-modal distribution which indicated there might be a few genes with a relatively large effect controlling this phenotype (Figure 1). Significant differences (*P* < 0.001) were found for all phenotypes among the lines (data not shown), as well as a wide range of variation for each of the fatty acids in the total oil concentration. The predominant fatty acid was oleic (C18:1), with the content varying from 59 to 82% with a mean value of 71.7% and a coefficient variation of 28.1. Oleic acid showed weak correlation with seed yield and C20:1 (*r* = 0.33 and 0.22 respectively), and was negatively correlated with C18:2 and C18:3 acids (*r* = −0.9 and −0.3 respectively). Linoleic acid (C18:2) was the second most abundant FA varying from 5 to 22% (mean value 13.1%) and a coefficient variation of 28.7. A weak correlation was found between C18:2 and seed weight (*r* = −0.2). The third most abundant FA in *B. napus* seed oil; linolenic acid, varied from 4.1 to 12% (mean 7.2) and a coefficient variation of 23. For most of the FAs and seed weight, a number of DH lines had higher or lower content of FAs than the two parents, which indicates a transgressive segregation in this population.

**Table 1:** **Means and ranges for fatty acids and seed weight of RIL324xRIL622 mapping population**.

	**C16:0**	**C18:0**	**C18:1**	**C18:2**	**C18:3**	**C20:1**	**Seed weight**
Min ±*SD*^a^	3.8 ± 0.15	1 ± 0.05	59.3 ± 2.1	6.6 ± 0.3	4.1 ± 0.7	1.3 ± 0.1	0.98 ± 0.5
Max ± *SD*	5.9 ± 0.55	3.6 ± 0.63	80.6 ± 0.8	22.3 ± 0.9	12 ± 0.3	1.9 ± 0.05	23.34
Mean ± *SD*	4.56 ± 0.55	1.80 ± 0.51	71.71 ± 4.8	13.17 ± 3.7	7.29 ± 1.7	1.48 ± 0.15	7.77 ± 4.93
RIL324	4.18 ± 0.17	1.40 ± 0.25	73.87 ± 2.68	9.55 ± 2.76	9.33 ± 1.54	1.3 ± 0.18	9.1 ± 3.7
RIL622	4.45 ± 0.29	1.58 ± 0.16	71.15 ± 1.77	14.5 ± 1.09	6.9 ± 0.65	1.35 ± 0.05	13.00 ± 5.7
CV (%)^b^	11.96	28.15	6.78	28.79	23.65	9.89	63.44

a *SD (standard deviation), calculated based on the measure values of seeds from the four replicated experimental blocks*.

b *coefficient of variation which was estimated as the ratio of the standard deviation to the mean of all accessions*.

**Table 2:** **Pearson correlations coefficient for the percentage contribution of each fatty acid to the total content of the TAG**.

	**C16:0**	**C18:0**	**C18:1**	**C18:2**	**C18:3**	**C20:1**	**Seed weight**
C16:0	1						
C18:0	−0.0162	1					
C18:1	−0.7091^**^	0.1661	1				
C18:2	0.6230^**^	−0.0299	−0.9477^**^	1			
C18:3	0.3351^**^	−0.6549^**^	−0.5833^**^	0.3220^**^	1		
C20:1	−0.1674	−0.3887^**^	0.2261^**^	−0.3879^**^	0.2957^**^	1	
Seed weight	−0.4926^**^	−0.1461	0.3395^**^	−0.2641^**^	−0.1634	−0.1546	1

*Trait pairs affecting fatty acid composition in the RIL324xRIL622 mapping population*.

** *significant at P < 0.01*.

### Skim genotyping by sequencing (GBS) pipeline results

Individual DH lines were sequenced with an average coverage of 1.2x (range 0.4x to 2.6x). The two parental individuals were sequenced with a coverage of 8.9x and 13.6x. Using these reads, SGSautoSNP called 91,205 SNPs between the two parental individuals, ranging from 84 (chrA08) to 9867 (chrC02) SNPs per chromosome (average: 3778), with an additional 19,422 SNPs identified on unplaced contigs. Low SNP number identified on chromosome A08 is due to no reads mapping for 29.36% of the chromosome in both parents. The SkimGBS pipeline used these SNPs to call 2,009,854 genotypes in total, an average of 29,556 (41.17%) SNPs per individual, ranging from 11,714 (16%) to 49,152 (68%) (Supplementary Table 1).

### Genome-wide association mapping identifies potential candidate genes regulating fatty acid composition in seeds of oilseed rape

**Figure 2:**
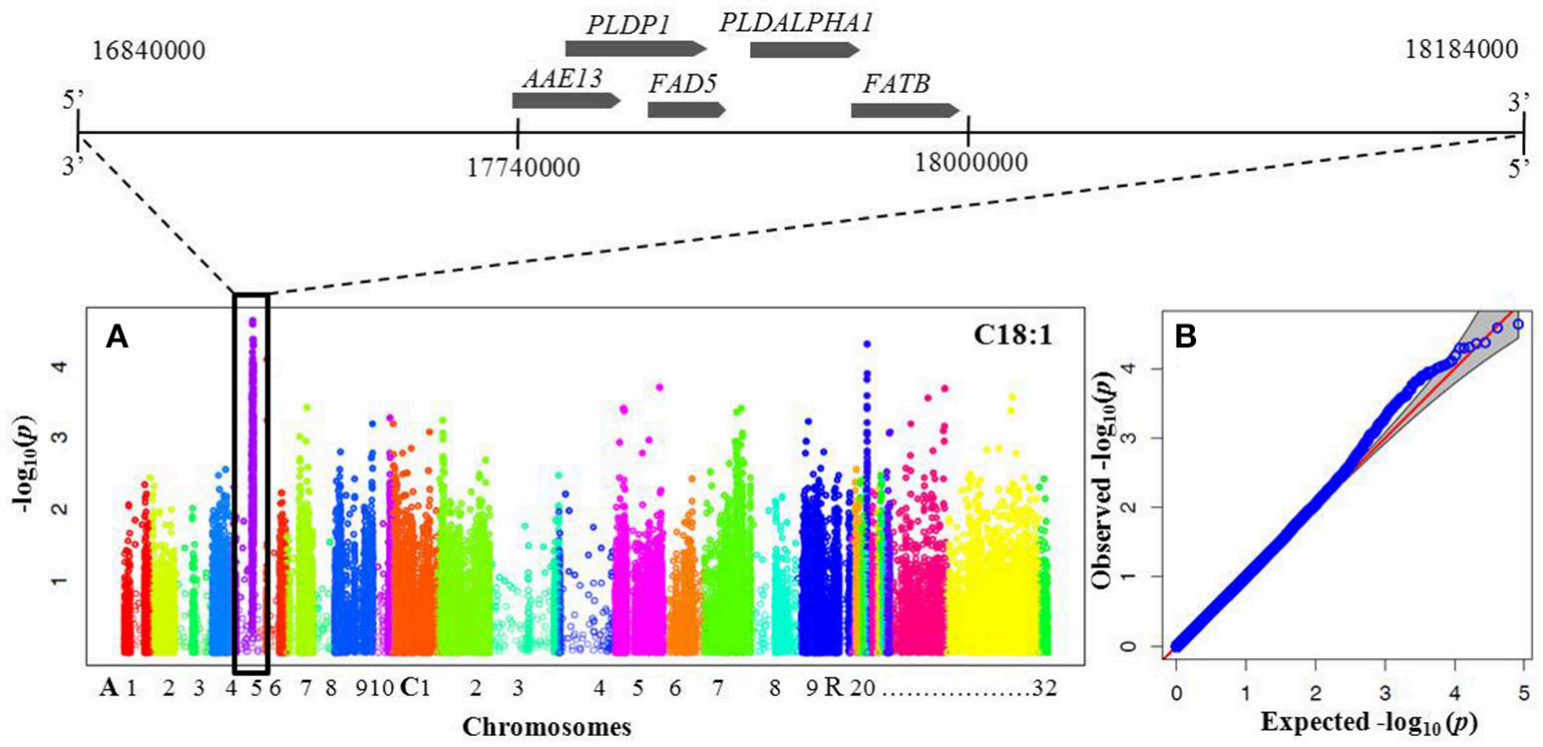
**Manhattan and quantile-quantile plots resulting from GWAS for oleic and linoleic fatty acids. (A)** Manhattan plot for oleic (C18:1) and linoleic (C18:2) fatty acid concentration. *X*-axis indicates SNP location along chromosomes depicted with different colors: A1–A10, C1–C9 and random chromosomes 20–32 (according to the *B. napus* Darmor v4.1 reference genome http://www.genoscope.cns.fr/brassicanapus/), *y*-axis is the log10(*p*) (*P*-value). Peak of significantly associated SNPs visible on chromosome A05. Genomic region surrounding a significant GWA peak for C18:1 and C18:2 FAs shown along the top with candidate genes known to be involved in lipid metabolism are listed in Table 3 and Supplementary Table 2. **(B)** Quantile-quantile (Q-Q) plot of *P*-values for oleic acid and linoleic fatty acid. The Y-axis is the observed negative base 10 logarithm of the *P*-values, and the X-axis is the expected observed negative base 10 logarithm of the *P*-values under the assumption that the *P*-values follow a uniform (0,1) distribution.

To elucidate the genetic architecture of FA composition in *B. napus* seeds, genome wide association analysis between FA and genotyped SNPs was performed using the panel of 60 DH mapping population lines. The association analysis with the 91,205 SNPs allowed identification of a peak of 34 significant associated SNPs with oleic acid and linoleic acids located between 17.2 and 18.2 Mbp on chromosome A05 (Figure 2). There were no significant associations with any of the other phenotypes tested.

**Figure 3:**
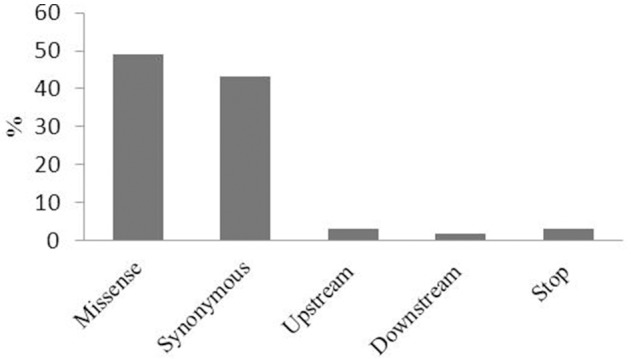
**The effect of GWAS associated SNP variants on genes**. The effect (missense, synonymous, upstream of a gene, downstream of a gene, stop) of GWAS associated SNP variants on the predicted genes represented as a percentage (%).

Physical mapping of the 34 most highly associated SNPs with oleic and linoleic FAs revealed that 16 of them were located in predicted *B. napus* genes whereas 18 were in regions with no predicted genes, transposable elements or repeated elements (Table 3). The analysis of the effect of the associated SNP variants revealed that 50% of them introduced a missense mutation in the predicted genes, whereas 37% of were synonymous and did not introduce any modifications in the amino acid sequence. A total of 12% of the mutations were found upstream and downstream of the coding sequence (Figure 3). Functional analysis of genes with the missense mutations allowed identification of promising candidate genes with an already known role in fatty acid regulation in model organisms (Table 3). One of the promising genes was an ortholog of Malonyl-CoA Synthase (*AAE13*) known to be involved in lipid metabolism in *A. thaliana*, with the G/T nucleotide change (UQSNP0001565) introducing N526K amino acid substitution. Regression analysis showed that *AAE13* associates with both oleic and linoleic FA content in seeds (*P* < 0.001) and explains their variability at 11.4 and 22.9%, respectively. *AAE13* associates also at lower level with stearic and eicosenoic acids (8.8 and 8.6%). Validation of the SNP showed it to be a true polymorphism (Table 4). A second promising gene encoding a Transducin/WD40 repeat-like superfamily protein (BnaA05g24090) showed C/G variation (UQSNP0001827) that causes a L348V amino acid substitution. Regression analysis with this gene also showed weak association with stearic acid (7.9% of variance) and strong with oleic (13.5% of variance) and linoleic acid (14.1%). An R323T amino acid substitution (UQSNP0001759) was found in the ortholog of a transcriptional regulator Pentatricopeptide repeat (PPR)-like gene (BnaA05g23930). The regression analysis with this gene also showed quite specific association with oleic (13.5% of variance) and linoleic fatty acid (24.3% of variance).

**Table 3:** **SNPs and candidate genes significantly associated with oleic acid and linolenic fatty acids**.

**Candidate gene**	**Nearest genes^c^**	**Position^d^**	**Allele**	**FDR adjusted *p*-value^f^**	**Annotation^g^**
BnaA05g22550^a^	BnaA05g22540, BnaA05g22610	17158994	C/T	0.415746	Cyclic Nucleotide binding factor 1 (*CNBT1*)
BnaA05g22710^a^	BnaA05g22680, Bna05g22740	17234557	T/A	0.415746	CBL-interacting serine/threonine-protein kinase1 (*CIPK1*)
EST *B. napus*^a^	Bna05g22950	17443201	A/G	0.415746	Nuclear fusion defective 2 (*NFD2*)
No gene prediction^b^	Bna05g23070	17479325	A/G	0.344113	Unknown
No gene prediction^a^, ^b^	Bna05g23070	17490748	G/T	0.415746^a^ 0.344113^b^	Unknown
No gene prediction^a^, ^b^	Bna05g23070, Bna05g23080	17491191	G/C	0.415746^a^ 0.344113^b^	Unknown
Repeats^a^, ^b^	Bna05g23110 *PLDP1* (Li M. et al., 2013) Bna05g23210	17529591	C/T	0.415746^*a*^0.344113^b^	Repeats
Repeats^a^, ^b^	Bna05g23110 *PLDP1* (Li M. et al., 2013) Bna05g23210	17539208	A/G	0.415746^a^ 0.344113^b^	Repeats
BnaA05g23370^a^, ^b^	Bna05g23360	17712452	A/C	0.415746^a^ 0.344113^b^	Small cysteine rich protein (*SCR-LIKE22*)
		17712458	G/T	0.415746^a^ 0.344113^b^	
EST *B. napus*^a^	Bna05g23360	17729054	A/G	0.415746	Unknown
BnaA05g23430^a^	Bna05g23360	17741099	C/G	0.415746	TRZ4, tRNAse
Bna05g23520^a^, ^b^	Bna05g23560	17777977	G/T	0.415746^a^ 0.344113^b^	Malonyl-CoA Synthase (*AAE13*)
EST *B. napus*^a^	Bna05g23360, Bna05g23560	17782780	A/G	0.415746	Unknown
EST *B. napus*^a^, ^b^	Bna05g23670 *FAD5*, BnaA05g23680 *FAD5* (Heilmann et al., 2004)	17889752	A/T	0.415746^a^ 0.344113^b^	UDP-Glycosyltransferase protein
Transposable element^a^, ^b^	BnaA05g23720, BnaA05g23740 *PLDALPHA1*	17920979	C/G	0.415746^a^ 0.344113^b^	TE
BnaA05g23770^a^, ^b^	BnaA05g23760, BnaA05g23790 *FATB* (Bonaventure et al., 2003)	17939130	T/G	0.415746^a^ 0.344113^b^	Fbox
BnaA05g23870^a^, ^b^	BnaA05g23830, BnaA05g23880	17986390	C/T	0.415746^a^ 0.344113^b^	Mitochondrial,glutaredoxin
BnaA05g23930^a^	BnaA05g23880	18018284	G/C	0.415746	Pentatricopeptide repeat (TPR)-like
BnaA05g23960^a^, ^b^	BnaA05g23880	18027977	A/G	0.415746^a^ 0.344113^b^	Unknown
		18027976	A/G	0.415746^a^ 0.344113^b^	
EST *B. napus*^a^	BnaA05g23880	18029252	A/G	0.415746	Unknown
EST *B. napus*^a^, ^b^	BnaA05g23880	18043857	A/T	0.415746^a^ 0.344113^b^	Unknown
Repeats^a^, ^b^	BnaA05g23880	18045729	A/T	0.415746^a^ 0.344113^b^	Repeats
Repeats^a^, ^b^	BnaA05g23880	18045779	A/T	0.415746^a^ 0.360659^b^	Repeats
Transposable element^a^, ^b^	BnaA05g23880	18046273	G/A	0.415746^a^ 0.344113^b^	TE
EST *B. napus*^a^, ^b^	BnaA05g23880	18057640	C/A	0.415746^a^ 0.344113^b^	UDP-Glycosyltransferase protein
EST *B. napus*^a^, ^b^	BnaA05g23880	18047838	G/A	0.415746^a^ 0.344113^b^	UDP-Glycosyltransferase protein
EST *B. napus*^a^, ^b^	BnaA05g23880	18047852	C/T	0.415746^a^ 0.344113^b^	UDP-Glycosyltransferase protein
BnaA05g24090^b^	BnaA05g24100	18140199	C/G	0.344113	Transducin/WD40 repeat-like superfamily protein
BnaA05g24230^a^, ^b^	BnaA05g24130	18182396^a^	G/T	0.415746	Legume lectin family protein
		18182414^a^, ^b^	C/T	0.415746 0.344113	
		18182687^b^	C/T	0.344113	

SNPs and potential candidate genes associated with oleic

a and b *linoleic fatty acid*.

c Nearest annotated genes within ~50 kb to associated SNP

d position in base pairs of associated SNP according to the B. napus Darmor v4.1 reference genome http://www.genoscope.cns.fr/brassicanapus/

f P-value of oleic and linoleic acid concentration

g *each candidate gene is annotated according to the B. napus Darmor v4.1 reference genome http://www.genoscope.cns.fr/brassicanapus/ and TAIR database https://www.arabidopsis.org/. Full list of genes available in Supplementary Table 2*.

**Table 4:** **Validation of SNPs in candidate genes**.

***B.napus*** **gene**	**Gene coordinates**	**Trait associated in GWAS**	**FDR adjusted** ***p*****-value**	**Annotation**	**Functional category**	**SNPs**	**SNPs position (Darmor v4)**	**Allele**	**SNP effect**	**Confirmed**	**Indels**	**Type**	**Position**
BnaA05g 23520	17777418–17781394	C18:1 C18:2	0.415746, 0.344113	*AAE13*	Lipid metabolism	UQSNP0001565	17777977	G/T	Missense variant	Confirmed	No		
BnaA05g 23670	17875337–17876795			*FAD5*	Lipid metabolism	UQSNP0001629	17876056	A/C	Synonymous variant	Amplified area did not cover SNP area	No		
						UQSNP0001630	17876071	T/C					
BnaA05g 23930	18017224–18019348	C18:1 C18:2	0.415746, 0.344113	PPR-like	Transcription	UQSNP0001759	18018284	G/C	Missense variant	Amplified area did not cover SNP area			
BnaA05g 24090	18138518–18143531		0.344113	WD40	Signal transduction	UQSNP0001827	18140199	C/G	Missense variant	Primers not specific			
BnaA05g 23740	17921857–17925521			*PLDALPHA1*	Lipid metabolism	UQSNP0001673	17922271	C/T	Missense variant	Confirmed	Yes	AT (insertion)	17922566
						UQSNP0001674	17923336	C/A	Synonymous variant	Confirmed		AGATTTGCTTTTCTTTTTAAAT (deletion)	17922836
						UQSNP0001675	17923519	T/G	Synonymous variant				
						UQSNP0001676	17923566	A/G	Synonymous variant				
						UQSNP0001677	17923726	A/G	Synonymous variant	Amplified area did not cover SNP area			
						UQSNP0001678	17924395	G/A	Synonymous variant				
						UQSNP0001679	17924465	G/T	Missense variant				
BnaA05g 23110	17520545–17528123			*PLDP1*	Lipid metabolism	UQSNP0001374	17521025	A/G	Missense variant	Confirmed		TTTAATTCT (deletion)	175520708
												AATATATAAGGTTATGATC (insertion)	17520803
						UQSNP0001377	17521925	A/G	Synonymous variant	No-Amplified area did not cover SNP area			
						UQSNP0001383	17523040	T/C					
						UQSNP0001384	17525851	C/A					
						UQSNP0001386	17526546	A/G					

**Figure 4:**
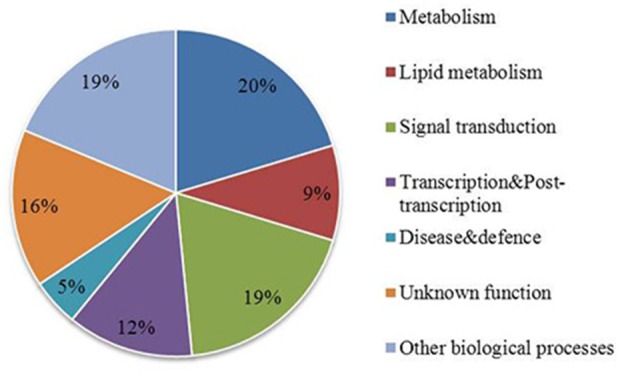
**Functional annotation of genes in the association region on chromosome A05**. Functional category annotation of genes significantly associated with oleic and linoleic fatty acid in GWAS analysis and their nearest annotated genes represented as a percentage.

The GWAS associated genes might not be directly involved in regulation of fatty acid biosynthesis but might be linked to the causal polymorphism in a nearby gene. The list of all the candidate genes located in the vicinity of associated SNPs is available in Supplementary Table 2. Functional annotation of associated genes and those located in their physical proximity showed that they are involved in various biological functions including metabolism, lipid metabolism, signal transduction, transcription and some are of unknown function (Figure 4). On the basis of functional annotation, it was inferred which candidate genes might be important in regulation of FA composition in seeds. One of the strong candidate genes, BnaA05g23790D was located 13 kb downstream of the associated SNP UQSNP0001703. This gene encodes an ortholog of Fatty acyl-ACP thioesterase B (*FATB*), a major enzyme involved in regulation of FA synthesis in seeds. The gene showed presence/absence variation (PAV) which was verified in the parental and individual DH lines of the mapping population using a PCR based assay and different combination of *FATB* gene specific primers (data not shown). The regression analysis showed that *FATB* associates with both oleic and linoleic seed fatty acid content (*P* < 0.001) and explains their variability at 16.8 and 30%, respectively. The estimate of regression coefficient for oleic acid was -0.0375 whereas for linoleic fatty acid 0.0643. This indicates that *FATB* regulates fatty acid content in the seeds of the mapping population in interaction with other genes. Another strong candidate gene (BnaA05g23670D) was located 13 kb upstream of the associated SNP UQSNP0001636 and encodes Palmitoyl-monogalactosyldiacylglycerol Delta7-desaturase FAD5 (*FAD5*). A/C variation between the parental lines of the mapping population in *FAD5* introduces a stop codon in the second exon of the gene. Interestingly, the parental lines did not harbor any SNP variation in the key FAD enzymes regulating oleic and linoleic acid, namely FAD2 and FAD3. Other candidate genes that are known to function in lipid metabolism in model plants included BnaA05g23740D, an ortholog of Phospholipase D alpha 1 (*PLDALPHA1*), located 1.3 kb downstream of associated SNP UQSNP0001669 and contained five synonymous and two missense mutations. Similarly, BnaA05g23110D, an ortholog of Phospholipase D p1 (*PLDP1*) was located 3 kb from UQSNP0001388 and harbored one missense variation between the parental lines. Validation of the SNP prediction using Sanger sequencing also identified an indel segregating in the parental and individual DH lines of the mapping population, in a haplotype with the other predicted SNPs in these 2 genes (Table 4). The regression analysis with the missense variants of both of the genes showed quite weak association with the phenotypes tested in this study. Genotype and phenotype data used for regression analysis is represented in Supplementary Table 4.

In order to validate our GWAS predicted SNPs we also undertook a genetic mapping approach and QTL analysis. Imputation using LD-kNNi raised the number of alleles from 2,272,337 to 4,030,644 alleles. MSTMap was able to place 43,484 out of 83,366 markers into linkage groups with more than one SNP. These linkage groups ranged from 420 to 7,037 SNPs with an average of 2,288. The 5 highest LOD scores and their positions in cM and bp are shown in Supplementary Table 5, and association was detected on the same region of chromosome A05, confirming the GWAS results, however with less resolution due to the lower number of markers mapped.

Together, the GWAS approach using high-resolution SkimGBS allowed identification of promising candidate genes affecting seed fatty acid content in *B. napus*.

## Discussion

Seed oil is a major energy reserve for seed germination and future seedling establishment, whereas for industrial purposes different fatty acid composition of oil is of great economic importance. In this study we analyzed the composition of FAs in *B. napus* DH progeny seeds derived from a cross between two recombinant parental lines and characterized SNPs in those lines. The GWAS approach using SkimGBS allowed us to identify candidate genes regulating oleic and linoleic fatty acid content in *B. napus* seeds.

We observed large variation in SNP coverage between the chromosomes of the parental lines, from 84 on chromosome A08 to 9,867 on chromosome C02. Whilst A08 still has the lowest number of SNPs, due to the short length of this chromosome it appears more extreme. Chromosomes A03, C03, and A05 have very similar percentages of identical alleles between the parental lines (88% of alleles are identical between parents on A08, 87% for A03, 82% for C03, and 77% for A05) (Supplementary Table 3). Overall the range of identical alleles is 48–88% per chromosome. The low marker density on chromosome A08 might be due to the missing read coverage along 29.36% (556,7248 bp) of the chromosome in both of the parental lines. Missing read coverage was observed in 7025 regions of chromosome A08 (average length of 792 bp) and it could be caused by differences between the genome of the parental lines and Darmor reference that we used to align the reads. We also observed that in one of the parental lines 786,414 bp in total (4.1% of chromosome A08) were not covered with reads when compared to the other parent. The missing reads were not in a single block but spread across 38,737 bp of smaller regions of an average length of 20 bp. This again could be due to the genomic differences between those lines. The low SNP density on A08 could also be due to relatively low level of genetic diversity between the parental lines as they both represent low erucic acid and low glucosinolates (double zero) winter type oilseed rape. Long and intensive breeding of double zero oilseed rape led to a restricted gene pool which reduces their genetic variation. Chromosome A08 could also represent genomic region of identity-by-descent that was not efficiently disrupted by recombination during selection (Browning and Browning, 2010).

The peak of significantly associated SNPs for oleic and linoleic fatty acid content in seeds was identified on chromosome A05, which corresponds with previous quantitative genetic studies reporting major QTL for those fatty acids on linkage group A05 (Smooker et al., 2011; Raman et al., 2013; Wang et al., 2015; Hu et al., 2006). Recently, integration of QTL and transcript abundance (eQTL) analysis in *B. rapa* also identified a QTL hotspot for polyunsaturated FAs on chromosome A05 (Basnet et al., 2016). The parental lines in our analysis did not harbor any genetic variation in any of the copies of desaturase FAD2, a key enzyme which regulates oleic acid content in *B. napus* seeds (Hu et al., 2006). Seed fatty acid biosynthesis is a complex trait that involves coordinated action of many genes (Baud and Lepiniec, 2010), therefore it is likely that variation of fatty acids in the studied *B. napus* mapping population is governed by an yet uncharacterized genetic network. One of the most promising candidates identified in the GWAS is BnaA05g23790D, a *FATB*. *FATB* showed presence/absence variation (PAV) between the parental lines. This variation would not be identified using traditional SNP analysis as reads from both parental lines are required to be mapped for SNP prediction. Thioesterases are key enzymes regulating fatty acid biosynthesis in seeds as they are involved in release of free fatty acids from acyl-ACP and their export from the plastids (Jones et al., 1995; Sun et al., 2014). The analysis of natural genetic variation in FA biosynthesis in *B. oleracea* revealed that the activity of the FATB enzyme was associated with a QTL on chromosome C5 (Barker et al., 2007). As a major determinant of fatty acid composition in seeds, FAT enzymes were also extensively studied and genetically modified in various oil crops, including *Ricinus communis, Macadamia tetraphylla*, and *Camelina sativa* (Sánchez-García et al., 2010; Moreno-Pérez et al., 2011; Rodríguez-Rodríguez et al., 2014; Kim et al., 2015). A search of the *B. napus* Darmor reference genome using the nucleotide sequence of our GWAS associated *FATB* gene identified three homolog, which share 100% nucleotide sequence identity with *FATB* on chromosomes A03 (BnaA03g47660D), C09 (BnaC09g30860D), and 99.87% identity on chromosome A07 (BnaA07g08340D). Interestingly, the identified genes were annotated as FATA enzymes. Most likely the annotation of BnaA05g23790D is incorrect, as a search of the Arabidopsis (TAIR) database also confirms closest sequence identity of BnaA05g23790D with the FATA gene. To avoid further confusion, we adhere to the publicly available annotation of BnaA05g23790D, and here we name it “*FATB*.” We did not identify any genetic variation between the parental lines in any of the three copies of *FATB*, which could imply that BnaA05g23790D copy of *FATB* is a functional which regulates fatty acid content in *B. napus* seeds. The regression analysis of *FATB* confirmed association of this gene with oleic and linoleic fatty acids, but also suggests that it is not the only gene responsible for fatty acid variation, but most likely interacts with other FA regulating genes. It is interesting that the parental line with the absence of the *FATB* copy contained less oleic acid and higher linoleic acid content when compared to the DH lines with the same genotype. DH lines with *FATB* absence showed an opposite phenotype, which could indicate that there was a high degree of transgression effect in the progeny lines. In the pre-breeding program, progeny lines derived from various crosses using the RIL324 parental line (higher oleic, low linoleic FA content) did not show phenotypic stability even in the F5 generation (personal communication, Plant Breeding and Acclimatization Institute-National Research Institute). This confirms that variation in oleic and linoleic acids in these genotypes are regulated by many genes rather than one single gene as well as possible environmental interaction on this phenotype. Further investigation is required to understand the role and mode of action of *FATB* in regulation of fatty acids in *B. napus* seeds.

In order to validate our GWAS results we also performed QTL analysis using the skimGBS data. The QTL mapping analysis identified the same genomic region, but the resolution was lower. The SkimGBS method uses low coverage data that is suitable for GWAS, but in QTL analysis missing reads lead to fewer markers that can be mapped which substantially affects resolution. The QTL approach is more suitable when no reference genome sequence is available, whereas GWAS can also be used when there is a reference genome and sequence based markers are available.

Our GWAS analysis led to the identification of several other promising candidate genes, one of which is an ortholog of *FAD5* (BnaA05g23670D), that belongs to the group of fatty acid desaturases. The role of *FAD5* was assigned to accumulation of one of the most abundant FAs present in leaves, known as Hexadeca 7,10,13-trienoic acid (16:3Δ7,10,13), and its role in seed oil biosynthesis is still not well understood (Heilmann et al., 2004). The analysis of lipid biosynthesis gene expression showed that *FAD5* had lower expression in *B. napus* seeds compared to leaves (Chen et al., 2015). In *B. rapa*, the expression of *FAD5* correlated with expression of *FAD2* and *FAD7* in seeds, and their expression QTLs (eQTLs) co-localized with QTLs for oleic acid, linoleic acid and other fatty acids in seeds (Basnet et al., 2016). These findings, together with our studies, imply a yet unknown interactive role of *FAD5* in the regulation of seed FA composition in *B. napus*. Other candidate genes found to be associated with oleic and linoleic FA content in this study include the predicted orthologous gene of *AAE13* (Malonyl-CoA Synthetase, Acyl Activating Enzyme 13, BnaA05g23520D) known to catalyze the formation of malonyl-CoA, a precursor for fatty acid synthesis and elongation (Chen et al., 2011; Guan and Nikolau, 2016). Our study allowed identification of a linoleic acid associated gene that encodes a Transducin/WD40 repeat-like superfamily protein (BnaA05g24090). In the genetic mapping study of *B. rapa*, WD-40 was reported as a candidate gene involved in the regulation of oleic acid (Basnet et al., 2016) and is known to play a role in TAG accumulation in *Drosophila melanogaster* (Häder et al., 2003). Another gene identified in our GWAS analysis, encoding an ortholog of a transcriptional regulator Pentatricopeptide repeat (PPR)-like gene (BnaA05g23930) in plants. PPR genes are known to be involved in plastid gene expression, and required for normal chloroplast development. Plastids are the site of biosynthesis of essential metabolites including fatty acids (Pyo et al., 2013). Mutation in this gene impairs proper seed development and produce seedling lethal phenotypes (Gutiérrez-Marcos et al., 2007).

## Conclusion

GWAS using an NGS SkimGBS approach allowed us to delineate a genomic region associated with oleic and linoleic acid content, which provides insight into the complex genetic architecture of biosynthesis of fatty acids in *B. napus* seeds. The presence of genes in the association hotspot known to regulate fatty acid biosynthesis confirms suitability of this approach. Future work will involve association analysis of the candidates genes with oleic and linoleic FA content. Understanding the mechanism of action and causal polymorphisms of these which genes will provide a better understanding of the role of those genes in regulation of fatty acid biosynthesis in seeds of this important oil-producing crop.

## Ethics statement

The authors declare that the study complies with the current laws of the countries (Australia and Poland) in which they were performed.

## Author contributions

IB-B and JBa supervised the project, LS developed DH mapping population used in the study, KG conducted phenotypic assessments and experiments, JBo conducted statistical analysis of data. KG analyzed the data with input from PB and JBa and wrote the manuscript. PB, DE, and JBa revised the manuscript. DE, PB, and JBa developed basic genetic resources and provided input into experimental design.

## Funding

This project, including a 3 month research visit by KG to the University of Western Australia, was supported by an EMBO short term fellowship. The authors would like to acknowledge funding support from the Australian Research Council (Projects LP130100925, LP140100537, and FT130100604) and Plant Breeding and Acclimatization Institute-National Research Institute, Poland.

### Conflict of interest statement

The authors declare that the research was conducted in the absence of any commercial or financial relationships that could be construed as a potential conflict of interest.

## Footnotes

^1^FAO, 2015. Available online at: http://www.fao.org/fileadmin/templates/est/COMM_MARKETS_MONITORING/Oilcrops/Documents/Food_outlook_oilseeds/FO_May_2015.pdf
